# Updated child protection legislation review with focus on Indigenous rights and data sharing, Canada

**DOI:** 10.24095/hpcdp.46.2.05

**Published:** 2026-02

The Government of Canada has released an updated version of *Provincial and Territorial Child Protection Legislation and Policy*,^1^ which offers a comprehensive overview of child protection laws across the country. Developed collaboratively by the Department of Justice Canada and the Public Health Agency of Canada (PHAC), the report highlights legislative changes from 2018 to 2023, with an added focus on Indigenous laws and information sharing for research or statistical purposes.

This updated report serves as a central resource for decision-makers, public health and child welfare professionals, researchers and others who may be interested in comparative analyses of legislation frameworks in child protection. The report complements PHAC’s related work on child maltreatment, including public health surveillance, the development of national child welfare data, applied research, and policy and programs. It responds to priorities set by PHAC’s Child Maltreatment Surveillance and Research Working Group, which also oversees the Canadian Child Welfare Information System, a national database that tracks children and youth in out-of-home care.

Key highlights of the report include:

- an overview of the *Act respecting First Nations, Inuit and Mtis children, youth and families*, including effects, governing principles and impacts;
- a snapshot of the age of eligibility for protection and extended services by jurisdiction;
- a summary of expanded support services for youth transitioning out of care, available up to 27 years of age in some regions;
- a review of mandatory reporting requirements with respect to child maltreatment;
- an examination of the rules related to sharing data for research or statistical purposes.

The report underscores Canada’s commitment to addressing child maltreatment, including child welfare, and advancing equity for Indigenous children and communities.
